# Analysis of Biofilm-Related Genes and Antifungal Susceptibility Pattern of Vaginal *Candida albicans* and Non-*Candida albicans* Species

**DOI:** 10.1155/2021/5598907

**Published:** 2021-05-28

**Authors:** Faezeh Mohammadi, Nima Hemmat, Zahra Bajalan, Amir Javadi

**Affiliations:** ^1^Cellular and Molecular Research Center, Research Institute for Prevention of Non-Communicable Disease, Qazvin University of Medical Sciences, Qazvin 34159-14595, Iran; ^2^Social Determinants of Health Research Center, Qazvin University of Medical Sciences, Qazvin 34159-14595, Iran; ^3^Department of Social Sciences, School of Medicine, Qazvin University of Medical Sciences, Qazvin 34159-14595, Iran

## Abstract

**Background:**

Vulvovaginal candidiasis caused by *Candida* species is a prevalent fungal infection among women. It is believed that the pathogenesis of *Candida* species is linked with the production of biofilm which is considered a virulence factor for this organism. The aim of this study was molecular identification, antifungal susceptibility, biomass quantification of biofilm, and detection of virulence markers of *Candida* species.

**Methods:**

We investigated the molecular identification of 70 vaginal isolates of *Candida* species, antifungal resistance to amphotericin B, fluconazole, itraconazole, and voriconazole according to CLSI M27-A3 and M27-S4, biofilm formation, and frequency analysis of biofilm-related ALS1, ALS3, and HWP1 genes.

**Results:**

Our findings showed that the most common yeast isolated from vaginal discharge was *C*. *albicans* (67%), followed by the non-*Candida albicans* species (33%). All *C*. *albicans* complex isolates were confirmed as *C*. *albicans* by HWP-PCR, and all isolates of the *C*. *glabrata* complex were revealed to be *C*. *glabrata sensu stricto* using the multiplex PCR method. FLC resistance was observed in 23.4% of *C*. *albicans* and 7.7% of *C*. *glabrata*. The resistance rate to ITC was found in 10.6% of *C*. *albicans*. The frequency of ALS1, ALS3, and HWP1 genes among *Candida* species was 67.1%, 80%, and 81.4%, respectively. Biofilm formation was observed in 54.3% of *Candida* species, and the highest frequency detected as a virulence factor was for the ALS3 gene (97.3%) in biofilm-forming species. *Discussion*. Our results showed the importance of molecular epidemiology studies, investigating antifungal susceptibility profiles, and understanding the role of biofilm-related virulence markers in the pathogenesis of *Candida* strains.

## 1. Introduction

Vulvovaginal candidiasis (VVC) is a common fungal infection in young women, and several factors such as age, diabetes, pregnancy, taking estrogen-containing birth control pills, antibacterial therapy, and the use of intrauterine devices have been identified as risk factors [1]. Vaginal candidiasis is associated with discharge, inflammation, burning, and redness. *Candida albicans* is the most important causative agent of VVC, followed by non-*Candida albicans* species [2]. The diagnosis of vulvovaginal candidiasis is often based on clinical signs and symptoms, and fluconazole is mostly used to treat *Candida* vaginitis. However, variable levels of resistance against antifungals may emerge in *Candida* species due to empirical administration or overuse of antimycotic agents [3, 4]. In recent decades, drug resistance has been increasing due to the overuse of antifungals [5, 6]. Therefore, understanding the antifungal susceptibility profiles of *Candida* species is key in guiding the selection of appropriate treatment for vulvovaginal candidiasis [6]. On the other hand, the pathogenicity of *Candida* species is increased with factors such as the ability to produce hyphae, binding and adhesion, extracellular enzyme production, and biofilm formation [7, 8]. The agglutinin-like sequence (ALS) gene family is the largest gene family known in *C*. *albicans* and is considered one of the important factors in the adhesion of the organism and the formation of biofilm [9]. The ALS gene family consists of several genes including the ALS1-ALS7 and ALS9 genes which play a role in the production of cell surface glycoproteins, leading to increased adhesion to host cells [10]. ALS1 and ALS3 genes affect adhesion to host epithelial cells and endothelial cells [11, 12]. Another protein affecting the adhesion and regulation of biofilm in *C*. *albicans* is the hyphal wall protein produced by the HWP1 gene [13].

HWP1 protein is a mannoprotein-linked glycosyl phosphatidylinositol that, similar to the protein encoded by the ALS family of genes, plays an important role in *Candida* adhesion [14].

Therefore, this protein is believed to have a significant link with the pathogenicity and virulence of *C*. *albicans* [14]. Several studies have shown that the HWP1-producing gene is expressed in the early stages of biofilm formation [15, 16]. The objective of this study was molecular identification, antifungal susceptibility profiling, biomass quantification of biofilm, and analysis of the distribution pattern of ALS1, ALS3, and HWP1 virulence genes of *Candida* isolates from VVC.

## 2. Materials and Methods

### 2.1. Sample Collection

A total of 135 genital clinical samples of the married women suspected of VVC were obtained from April to October 2020 who referred to the Kowsar gynecology clinic in Qazvin, Iran. The consent form was obtained from all participants. Pregnancy, douching history of less than past 48 hours, lack of patient contentment, and history of recent consumption of antimicrobials were considered exclusion criteria. The study was approved by the Ethics Committee of Qazvin University of Medical Sciences (IR.QUMS.REC.1397.085). A midwife was in charge of taking the vaginal specimens using sterile swabs, followed by rapid delivery of samples to the Qazvin Medical School mycology laboratory. The Sabouraud dextrose agar medium (SDA, Difco) was used to culture the vaginal sample which was later incubated at 30°C. The phenotypic test was performed using the chromogenic medium of CHROMagar Candida (CHROMagar Company, Paris, France).

### 2.2. DNA Extraction and ITS-PCR

Genomic DNA was extracted using glass beads and phenol : chloroform : isoamyl alcohol (25 : 24 : 1) [17]. Briefly, the ITS1-5.8S-ITS2 region of *Candida* species was amplified using universal fungal primers ITS1 (forward 5′-TCCTCCGCTTATTGATAT GC-3′) and ITS4 (reverse 5′-TCCTCCGCTTATTGATAT GC-3′) [15]. The PCR mixture contained 12.5 *μ*L of Taq Mix Red 2X, 30 pmol of each primer, and 3 *μ*L of the DNA sample in a final volume of 25 *μ*L. Amplification was performed as follows: initial denaturation at 94°C for 5 min, followed by 35 cycles of 30 s at 94°C, 45 s at 55°C, 1 min at 72°C, and a final extension of 7 min at 72°C. The PCR products were analyzed by agarose gel electrophoresis.

### 2.3. PCR-RFLP Analysis

PCR products were digested with the *MspI* enzyme (Thermo Fisher Scientific, Lithuania). The 30 *μ*L reaction mixture contained 1 *μ*L of the *MspI* enzyme, 2 *μ*L of the buffer, 10 *μ*L of the PCR product, and sterile distilled water to the final volume, followed by incubation at 37°C for 3 h. The PCR*-*RFLP products were analyzed by agarose gel electrophoresis.

### 2.4. Hyphal Wall Protein 1 (HWP1) Gene and Multiplex Polymerase Chain Reaction

All *C*. *albicans* isolates were confirmed by PCR amplification of the HWP1 gene, using CR-f and CR-r primer pairs (Table 1). Conditions for HWP-PCR were as follows: initial denaturation at 95°C for 5 min, followed by 35 cycles of 45 s at 94°C, 40 s at 58°C, 55 s at 72°C, and a final extension of 10 min at 72°C. The identification of the *C*. *glabrata* complex including *C*. *glabrata sensu stricto*, *C*. *bracarensis*, and *C*. *nivariensis* was achieved using the universal reverse primer (UNI-5.8S), GLA-f, NIV-f, and BRA-f (Table 1) [16]. In brief, the reaction mixture contained 12.5 *μ*L of Taq Mix Red 2X, 0.2 *μ*M of each of the four primers, and 10 ng of genomic DNA. Multiplex amplification was performed as follows: initial denaturation at 95°C for 5 min, followed by 34 cycles of 30 s at 94°C, 40 s at 60°C, 50 s at 72°C, and a final extension of 10 min at 72°C.

### 2.5. Antifungal Susceptibility Test

Susceptibility assay was performed in 96-well plates by following the M27-A3 and M27-S4 guidelines of the Clinical and Laboratory Standards Institute [18] for *in vitro* testing [19, 20].

The stock solutions of amphotericin B (AMB), fluconazole (FLC), itraconazole (ITC), and voriconazole (VRC) (Sigma-Aldrich Chemical Corporation, St. Louis, MO, USA) were prepared in the RPMI-1640 medium (pH = 7.0). Briefly, 100 *μ*L of the RPMI-1640 medium (Sigma, Germany) and serial dilutions from AMB, ITC, and VRC, ranging from 0.032 to 16 *μ*g/mL, and from FLC, ranging from 0.06 to 64 *μ*g/mL, were poured in each well. The yeast suspension equal to 0.5 McFarland (1–5 × 10^6^ cells/mL) was prepared of fresh colonies and diluted at 1 : 1000 with RPMI (1–5 × 10^3^ cells/mL). The final suspensions (100 *μ*L) were added to each well, except the control negative well. Minimum inhibitory concentration (MIC) was determined after 48 h of incubation at 35°C. *Candida parapsilosis* (ATCC 22019) and *Candida krusei* (ATCC 6258) were used as quality controls. Antifungal susceptibility testing was performed in duplicate.

### 2.6. Biofilm Formation

The biofilm production was evaluated using a 1% crystal violet (CV) solution *in vitro*. In brief, the yeast cell suspension and Sabouraud dextrose broth (SDB), supplemented with 60 g of glucose, were inoculated into wells of 96-well microplates [21]. After incubation at 35°C for 24 hours, the wells were washed three times with sterile PBS and stained with crystal violet for 15 min. Finally, fixation was performed using ethanol-acetone (80 : 20, *v*/*v*). The absorbance values were determined using an ELISA reader at 590 nm. Biofilm formation assay was repeated three times for each strain.

### 2.7. Scanning Electron Microscopy (SEM)

The highest levels of biofilm-forming species among the tested ones were selected according to the method for SEM image analysis. After incubation at 35°C for 24 hours, the planktonic cells were removed and biofilm cells were fixed with glutaraldehyde. After incubation, the biofilms were dehydrated with various dilutions of ethanol (20%, 40%, 60%, 80%, and 100%). Finally, a network of dense hyphal and yeast cells of *Candida* biofilms was examined with scanning electron microscopy (SEM) [22].

### 2.8. Detection of Virulence Gene Markers

Extracted DNA was used for amplification of HWP1, ALS1, and ALS3 as virulence genes. The primer sequences are revealed in Table 1. The amplification was performed in a 25 *μ*L reaction mixture containing Taq Mix Red 2X, 10 mM of each primer, 10 ng of template DNA, and nuclease-free water up to 25 *μ*L. The PCR reactions were as follows: initial denaturation at 95°C for 5 min, followed by 32 cycles of 1 min at 95°C, 1 min at 55°C, 1 min at 72°C, and a final extension of 10 min at 72°C. The products were visualized by gel electrophoresis.

## 3. Results

A total of 135 vaginal clinical samples of married women suspected of VVC were examined. The mean age of the patients was 31 ± 6.5 years (range: 19 to 46 years). Direct examination and culture results showed that out of 135 suspected women included in the present study, 70 (51.8%) were positive for VVC. Among women who suffered from VVC, the frequency of clinical symptoms included white discharge (83%), itching (77%), inflammation (60%), and irritation (54.3%), respectively.

### 3.1. *Candida* Species Identification

**Figure 1:**
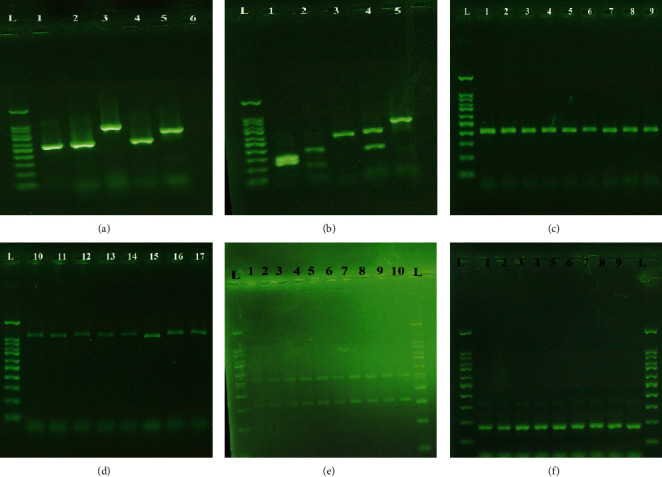
Lane L: 100 bp DNA size marker. (a) Agarose gel electrophoresis of ITS-PCR products. Lanes 1-5: *C*. *albicans*, *C*. *tropicalis*, *C*. *glabrata*, *C*. *parapsilosis*, and *C*. *kefyr*. Lane 6: negative control. (b) Agarose gel electrophoresis of *MspI*-PCR of vaginal *Candida* species. Lanes 1-5: *C*. *albicans*, *C*. *tropicalis*, *C*. *parapsilosis*, *C*. *glabrata*, and *C*. *kefyr*. (c) Multiplex PCR method for the *C*. *glabrata* complex. Lanes 1-9: *C*. *glabrata*. (d) Agarose gel electrophoresis of HWP-PCR. Lanes 10-17: *C*. *albicans*. (e) Multiplex PCR products of ALS1 (319 bp) and HWP1 (503 bp) gene strains of *Candida* species. (f) Agarose gel electrophoresis of PCR for the ALS3 gene (185 bp) of *Candida* species.

The PCR amplification of the ITS region of all clinical isolates is shown in Figure 1. The *MspI*-PCR analysis indicated that *C*. *albicans* was the most prevalent isolated species (47, 67%), followed by *C*. *glabrata* (13, 18.5%), *C*. *kefyr* (5, 7%), *C*. *tropicalis* (3, 4.2%), and *C*. *parapsilosis* (2, 2.8%) (Figure 1).

### 3.2. *Candida albicans* and *C*. *glabrata* Complex Identification

The multiplex PCR for the identification of 13 isolates of the *Candida glabrata* complex showed that the amplified products were solely a 397 bp sequence comparable with *C*. *glabrata sensu stricto*. None of the isolates belonged to *C*. *nivariensis* and *C*. *bracarensis* (Figure 1). Furthermore, the molecular identification using the HWP1 gene demonstrated that the PCR product of all isolates was a 1000 bp sequence belonging to *C*. *albicans sensu stricto* (Figure 1).

### 3.3. Antifungal Susceptibility Profile of Vaginal Isolates of *Candida* species

The antifungal susceptibility profile of 47 *C*. *albicans* isolates recovered from VVC showed that all isolates were susceptible to AMB and VRC, whereas eleven isolates (23.4%) were resistant to FLC (MIC ≥ 64 *μ*g/mL), and six isolates (12.8%) were found to be susceptible-dose dependent (SDD). Furthermore, five isolates (10.6%) demonstrated the highest MIC to ITC (MIC ≥ 1 *μ*g/mL). Among non-*Candida albicans* species, all *C*. *kefyr*, *C*. *tropicalis*, and *C*. *parapsilosis* were susceptible to AMB, FLC, ITC, and VRC, whereas the resistance rate of *C*. *glabrata* to FLC was 7.7%. All *C*. *glabrata* isolates were susceptible to AMB, ITC, and VRC. The MIC values and geometric means for FLC, ITC, and VRC are shown in Table 2.

### 3.4. Biofilm Formation of *Candida* Species

The rate of biofilm production was detected in 54.3% of vaginal isolates of *Candida* with a mean Abs of 0.36 ± 0.31. The biofilm production at weak (+), moderate (++), and strong (+++) levels was found in 20%, 11.4%, and 21.4% of *Candida* isolated from VVC, respectively. In addition, biofilm formation was observed in 26.3% and 13.2% of FLC- and ITC-resistant isolates, respectively.

### 3.5. Detection of Virulence Gene Markers

**Figure 2:**
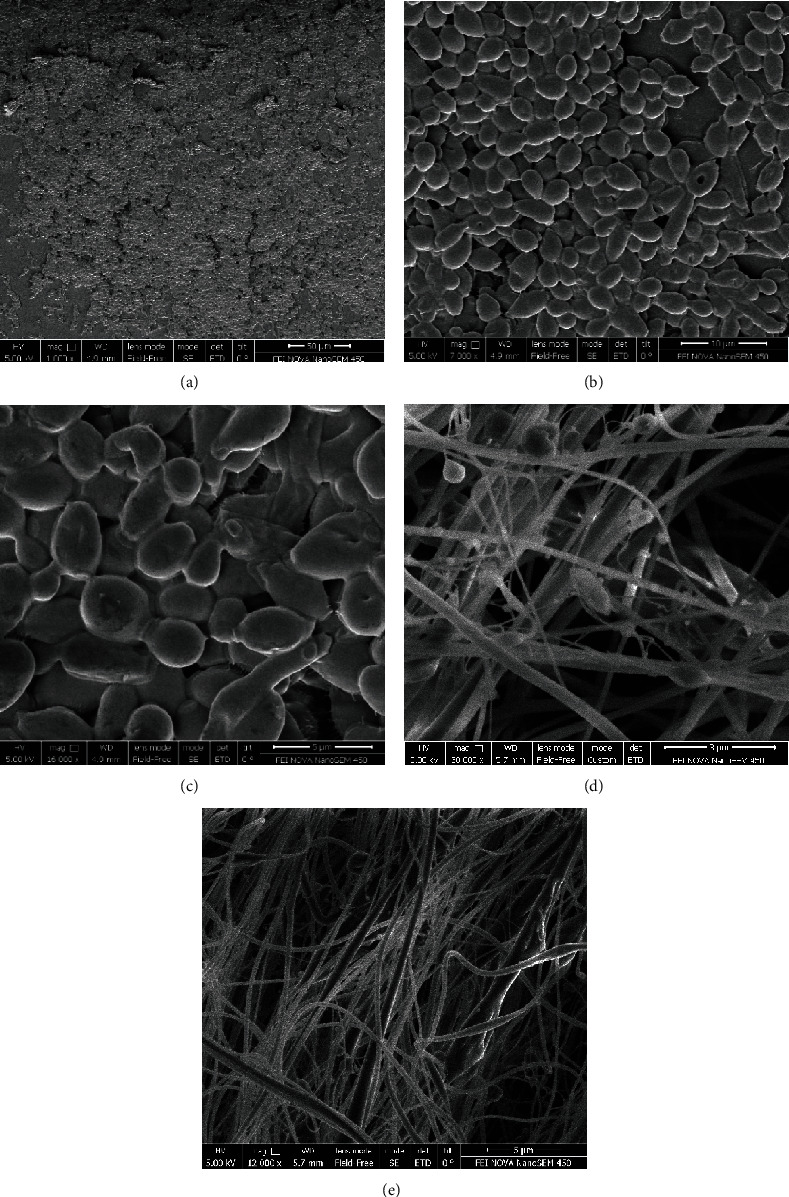
Scanning electron microscopy (SEM) images of the network of dense yeast cells and hyphal in biofilm-forming strains of *C*. *albicans* isolated from vaginal samples.

The frequency of ALS1 and HWP1 genes determined by PCR assay was as follows: *C*. *albicans* (*n* = 37/47; 78.7% and 42/47; 89.4%), *C*. *glabrata* (*n* = 7/13; 53.8% and 8/13; 61.5%), *C*. *kefyr* (*n* = 2/5; 40% and 4/5; 80%), and *C*. *tropicalis* (*n* = 1/3; 33.3% and 2/3; 66.7%) (Figure 1). The ALS1 gene was not detected in *C*. *parapsilosis*, whereas the HWP1 gene was observed in 50% of *C*. *parapsilosis*. The prevalence of the ALS3 gene was in *C*. *albicans* (*n* = 43/47; 91.5%), *C*. *glabrata* (*n* = 6/13; 46.2%), *C*. *kefyr* (*n* = 3/5; 60%), *C*. *tropicalis* (*n* = 3/3; 100%), and *C*. *parapsilosis* (*n* = 1/2; 50%) (Figure 1). The simultaneous presence of ALS1, ALS3, and HWP1 genes was observed in 27% and 15.4% of fluconazole- and itraconazole-resistant biofilm-producing species, respectively (Table 3). The result of SEM of the network of dense hyphal by *C*. *albicans* is shown in Figure 2. The simultaneous presence of ALS1, ALS3, and HWP1 genes was found in 95% of studied strains. Comparison of the frequency of virulence genes with the phenotypic biofilm test revealed the presence of ALS1, ALS3, and HWP1 genes in 73%, 97.3%, and 89.2% of biofilm-producing *Candida* species, respectively.

## 4. Discussion

It is reported that a quarter of women are exposed to vaginal candidiasis during their lifetime [23]. In recent years, there have been reports of non-*Candida albicans* agents isolated from women with VVC. Our findings showed that the most common yeast isolated from vaginal discharge was *C*. *albicans* (67%), followed by *C*. *glabrata* (18.5%), *C*. *kefyr* (7%), *C*. *tropicalis* (4.2%), and *C*. *parapsilosis* (2.8%) The distribution of *Candida* isolates in our study was similar to other studies [24–28]. In our study, all the *C*. *albicans* complex isolates were confirmed as *C*. *albicans* based on the presence of a 1000 bp band, whereas no band was detected for either *C*. *dubliniensis* or *C*. *africana*. Shan et al. reported that the *C*. *albicans* complex isolated from vaginal samples included 98.5% of *C*. *albicans* and 1.5% of *C*. *africana* by HWP-PCR. Meanwhile, the authors failed to demonstrate *C*. *dubliniensis* in their samples [29]. Mucci et al. employed CR primers and indicated that the prevalence of *C*. *albicans* and *C*. *dubliniensis* in the samples obtained from vulvovaginal candidiasis was 94.7% and 2.6%, respectively [30]. In our study, the multiplex PCR method showed that 13 strains of the vaginal *C*. *glabrata* complex were identified as *C*. *glabrata sensu stricto*. Li et al. reported that out of 301 *C*. *glabrata* complex strains isolated from Chinese women with VVC, 97.3%, 2.3%, and 0.3% were identified as *C*. *glabrata*, *C*. *nivariensis*, and *C*. *bracarensis*, respectively [31].

The antifungal susceptibility testing of all *Candida* isolates in the current study demonstrated that AMB and VRC had excellent antifungal activity against all of the species of *Candida* evaluated. These findings are in agreement with the data of Montagna et al. from Italy [32], Tseng et al. from Taiwan [33], and Ganeshkumar et al. from India [34]. Lovero et al. showed that Etest is a reliable method for determining the susceptibility of *Candida* species to L-AMB [35]. In Greece, the antifungal susceptibility profile has shown that *Candida* isolates from patients with vulvovaginal candidiasis to AMB and VRC were 0.2% and 1.4%, respectively [36]. In addition, Ghaddar et al. revealed that 97.5% of *C*. *albicans* isolates from VVC patients were resistant to VRC [37]. Our findings demonstrated that 23.4% and 10.6% of *C*. *albicans* isolates were resistant to FLC and ITC, respectively. The fluconazole resistance rate was reported from Belgium, Ethiopia, and Taiwan at 21%, 53.7%, and 14.7%, respectively [25, 33, 38]. Similarly, Adjapong et al. reported that 10% of *C*. *albicans* isolates from VVC patients were resistant to ITC [39]. Edrees et al. showed that 29.6% of *C*. *albicans* isolates from VVC patients were resistant to ITC [40]. In addition, our findings showed that the resistance rate of *C*. *glabrata* to fluconazole was 7.7%. Miranda-Cadena et al. showed that the resistance rate to FLC and ITC in *C*. *glabrata* was 7.7% and 6.1%, respectively [41]. In Iowa, 15.2% of *C*. *glabrata* isolates from VVC were fluconazole resistant [42]. Biofilm formation, as a virulence factor, plays an important role in the pathogenesis and binding of *Candida* species to host cells [43]. In our study, the rate of biofilm production was detected in 54.3% of VVC-associated *Candida* species. Biofilm formation was also found in 26.3% and 13.2% of FLC- and ITC-resistant isolates, respectively. Studies show that *Candida* species can help to reduce drug penetration and increase antifungal resistance by forming a dense network of pseudohyphae in biofilms [44, 45]. The results of several studies showed that the ALS3 and ALS1 genes of the ALS family as well as the HWP1 gene play an important role in the *Candida* species adhesion and biofilm formation process [46]. Our results revealed that the highest frequency for the genes tested in the present study was obtained for the HWP1 gene (81.4%) of *Candida* species isolated from VVC. ALS3 and HWP1 genes were detected in all isolates of *Candida* spp. studied, while the ALS1 gene was not detected in *C*. *parapsilosis*.

Ardehali et al. reported that the highest virulence factor identified was the HWP1 gene, with a frequency of 95% in *C*. *albicans* isolated from blood and urinary infections. In their study, the presence of ALS3 and HWP1 genes in *C*. *glabrata* was higher than that of the ALS1 gene. In addition, the ALS1 gene was not detected in *C*. *tropicalis* [47]. Shrief et al. reported a frequency of 77% for the HWP1 gene and 65% for the ALS1 gene of *C*. *albicans* isolated from blood and urine cultures. They also described that 58% of *C*. *albicans* were capable of biofilm production [48]. The prevalence of the ALS1 and HWP1 genes was detected in 48% and 3.9% of the strains isolated from vaginal samples in Turkish women, respectively [49].

A study on Brazilian women showed that the frequency of ALS1 and HWP1 genes in the *C*. *albicans* collected from vaginal discharge was 73.6% and 21%, respectively [50]. Differences in the type of clinical samples and techniques used for diagnosis may cause variations in the frequency of genes in various studies. There are many reports that have been concerned with the expression of ALS and HWP genes. Cheng et al. indicated that the ALS1 and ALS3 genes were expressed in both the clinical vaginal discharge and the experimental model system for vaginal candidiasis [51]. The results of our study and several other studies have shown that the frequency of the presence of these genes and their expression in clinical isolates can indicate the role of these genetic markers in promoting the adhesion and biofilm formation in *Candida* species, especially *C*. *albicans*. Moreover, the simultaneous presence of ALS1, ALS3, and HWP1 genes plays a role in enhancing the synergistic effect on the performance of individual genes involved [13, 52]. In our study, the frequency of the ALS3 gene was found in 94.7% of biofilm-positive species which was higher than the other genes examined. Furthermore, the simultaneous presence of ALS1, ALS3, and HWP1 genes in biofilm-producing *Candida* species was 52.8%. In Egypt, the biofilm capacity was observed in 58% of *Candida albicans* isolates from the chemotherapy patients. Also, the prevalence of ALS1 and HWP1 genes among the biofilm-positive *C*. *albicans* was 56.9% [48]. Inci et al. showed the presence of ALS1 and HWP1 genes in 83.1% and 11.3%, respectively, of biofilm-producing isolates [49].

## 5. Conclusion

In our study, *C*. *albicans* showed the highest frequency for ALS1, ALS3, and HWP1 genes. This high prevalence rate for virulence factors is significant in *C*. *albicans* vaginal specimens. It is of great concern that several studies have shown that *Candida* biofilm formation may be involved in resistance to antifungals. Since biofilm formation affects the process of vulvovaginal candidiasis infection, it is important to understand the role of virulence factors in the pathogenesis of *Candida* species.

## Figures and Tables

**Table 1:** The primer sequences for identification of the *C*. *albicans* complex, *C*. *glabrata* complex, and virulence gene markers.

Primer name	Sequence (5′⟶3′)	Species identified and product size (bp)
CR-f	GCTACCACTTCAGAATCATCATC	*C*. *albicans*: 1000
CR-r	GCACCTTCAGTCGTAGAGACG	*C*. *dubliniensis*: 569
		*C*. *africana*: 700
UNI-5.8S	ACCAGAGGGCGCAATGTG	*C*. *glabrata*: 397
GLA-f	CGGTTGGTGGGTGTTCTGC	*C*. *nivariensis*: 293
NIV-f	AGGGAGGAGTTTGTATCTTTCAAC	*C*. *bracarensis*: 233
BRA-f	GGGACGGTAAGTCTCCCG	
ALS1	ACCAGAAGAAACAGCAGGTG	319
	GACTAGTGAACCAACAAATACCAG	
ALS3	CCAAGTGTTCCAACAACTGAA	185
	GAACCGGTTGTTGCTATGGT	
HWP1	ATGACTCCAGCTGGTTC	503
	TAGATCAAGAATGCAGC	

**Table 2:** Antifungal susceptibility pattern of 70 *Candida* species isolated from vulvovaginal candidiasis.

			MICs^1^ (*μ*g/mL)		
Species	Antifungal	MIC range	MIC50	MIC90	GM^2^
*C*. *albicans*	AMB^3^	0.032-0.5	0.25	0.5	0.155
	FLC^4^	0.062-64	0.5	16	1.05
	ITC^5^	0.062-4	0.125	0.25	0.112
	VRC^6^	0.016-0.125	0.062	0.125	0.059
*C*. *glabrata*	AMB	0.032-0.25	0.125	0.25	0.091
	FLC	0.032-64	0.25	1	0.073
	ITC	0.016-0.125	0.032	0.125	0.043
	VRC	0.032-0.125	0.062	0.125	0.292
*C*. *kefyr*	AMB	0.016-0.032	0.062	0.125	0.054
	FLC	0.032-0.25	0.125	0.25	0.109
	ITC	0.032-0.125	0.062	0.125	0.071
	VRC	0.016-0.125	0.032	0.125	0.048
*C*. *tropicalis*	AMB	0.032-0.016	0.032	0.06	0.031
	FLC	0.125-0.5	0.25	0.5	0.25
	ITC	0.032-0.125	0.125	0.125	0.08
	VRC	0.032-0.016	0.032	0.032	0.025
*C*. *parapsilosis*	AMB	0.032-0.032	0.032	0.032	0.032
	FLC	0.5-0.5	0.5	0.5	0.5
	ITC	0.032-0.032	0.032	0.032	0.032
	VRC	0.016-0.016	0.016	0.016	0.016

^1^MIC: minimum inhibitory concentration. ^2^GM: geometric mean. ^3^AMB: amphotericin B. ^4^FLC: fluconazole. ^5^ITC: itraconazole. ^6^VRC: voriconazole.

**Table 3:** Presence of virulence genes in biofilm-producing *Candida* species resistant to fluconazole and itraconazole.

Presence of virulence genes	Biofilm-producing species and FLC^1^	Biofilm-producing species and ITC^2^
	Resistant	Resistant
ALS1	7 (26%)	5 (18.5%)
ALS3	9 (25%)	5 (14%)
HWP1	10 (29.4%)	4 (11.8%)
ALS1, ALS3, and HWP1	7 (27%)	4 (15.4%)

^1^FLC: fluconazole. ^2^ITC: itraconazole.

## Data Availability

The data used to support the findings of this study are available from the corresponding author upon request.
